# Subzero saline chilling with or without prechilling in icy water improved chilling efficiency and meat tenderness of broiler carcasses

**DOI:** 10.1016/j.psj.2023.103070

**Published:** 2023-08-26

**Authors:** K. Kawamura, D. Ma, A. Pereira, D.U. Ahn, D.M. Kim, I. Kang

**Affiliations:** ⁎ Department of Animal Science, California Polytechnic State University, San Luis Obispo, CA 93407, USA; † Department of Food Science & Nutrition, California Polytechnic State University, San Luis Obispo, CA 93407, USA; ‡ Department of Animal Science, Iowa State University, Ames, IA 50011, USA; § Department of Biochemistry, California Polytechnic State University, San Luis Obispo, CA 93407, USA

**Keywords:** subzero saline chilling, water immersion chilling, chilling efficiency, shear force, breast color

## Abstract

Freshly slaughtered carcasses need to be chilled to improve product quality, meat safety, and processing efficiency. This research investigated the effect of subzero saline chilling (**SSC**) on broiler carcasses with or without prechilling in icy water. Water immersion chilling at 0.5°C (**WIC**) or SSC at 4% NaCl/−2.41°C (SSC) was a major chilling step. For the combination of pre- and postchilling, the warm water immersion chilling (**WWIC**) at 10°C was used as prechilling and the WIC as postchilling (WWIC-WIC), and WIC was used as prechilling and the SSC as postchilling (WIC-SSC). The internal temperature of breast fillets was monitored during chilling. Carcasses in a prechiller were transported to a postchiller when their internal temperature reached 15°C. Chilling was completed when the carcass temperature reached 4.4°C or below, and breast fillets were harvested at 3-h postmortem to measure the pH and sarcomere length. Color (L*, a*, and b*) values were evaluated on both breast skin and skinless breast surfaces. Meat tenderness was evaluated using the breast fillets after overnight storage and cooking to an internal temperature of 76°C. The carcasses in the SSC and WIC-SSC showed shorter chilling times (85–91 min) than those (100–144 min) of WIC and WWIC-WIC. A higher chilling yield was observed for the carcasses in WIC-SSC, and a lower cooking yield was seen for the carcasses in WWIC-WIC than other chilling methods (*P* < 0.05). The breast fillets of broilers in the SSC and WIC-SSC showed lower shear forces and longer sarcomere length than the WIC and WWIC-WIC. No difference was found for L* and a* values, while lower b* value was observed in the SSC than the other chilling methods (*P* < 0.05). Based on these results, chilling of broiler carcasses in the SSC (4% NaCl/−2.41°C) with or without prechilling in WIC at 0.5°C significantly improved chilling efficiency and meat tenderness, with minor color changes on carcasses.

## INTRODUCTION

Poultry carcasses are required to be chilled after evisceration for the prevention of bacterial growth and quality loss (Huezo et al., 2007; Sams and McKee, 2010). Many poultry plants in the United States use single or 2 stages of chiller such as prechiller and/or postchiller (or main chiller). The main purpose of the prechiller is to allow carcasses to have a gradual chilling with some washing effects. The water temperature of the prechiller is about 7°C to 12°C, where carcasses are allowed to be chilled for 10 to 15 min with 2 to 4% water absorptions during the prechill (Sams and McKee, 2010).

Currently, a proprietary chilling method has been developed using subzero saline solutions (Kang, 2019; Husband, 1993). The effects of 6 chilling solutions (0, 1, 2, 3, 4, and 8% NaCl) were initially evaluated in the chilling temperatures from 0.5°C to 5.08°C (Metheny et al., 2019). Results indicated that both chilling efficiency and meat tenderness were proportionally increased as the chilling temperature decreased and the saline concentration increased, with no significant difference in meat tenderness between 4% NaCl/−2.41°C and 8% NaCl/−2.41°C solutions. Recently, broilers chilled in 4% NaCl/−2.41°C was reported to reduce bacterial counts, which was similarly observed in turkey carcasses too (Lee et al., 2020; Kang et al., 2021). Additionally, the combination of hot water spray and subzero saline chilling (**SSC**) further improved bacterial decontamination on broiler carcasses (Kang et al., 2022).

Sams and McKee (2010) indicated that warm carcasses (∼38°C) in prechiller absorb water as the skin lipids are quite fluid, whereas less warm carcasses (30°C–35°C) in postchiller seal the absorbed water as the tissue lipids solidify in the cold temperature at 1°C. No research has been conducted for the effects of 2 chilling steps with water immersion chilling (**WIC**) at 0.5°C as a prechill and SSC at −2.41°C as a postchill, except the shear force evaluation by Metheny et al. (2019). The objective of this study was to further investigate the effects of SSC on broiler carcasses with or without WIC at 0.5°C as a prechill.

## MATERIALS AND METHODS

All procedures were approved by the Institutional Animal Care and Use Committee of California Polytechnic State University (Protocol #2010).

### Preparation of Broiler Birds, Control Solutions, and Subzero Saline Solution

Seventy-two commercial broilers (Ross 708, 6-wk-old with 2.5–3.7 kg live weights) were transferred from a local commercial plant to the Meat Processing Center at California Polytechnic State University (Cal Poly) for processing. A control solution (0.5°C) was prepared with ice, and the target temperature was maintained in a refrigerated room with continuous agitation using a submersible pump (4E-34N, SupplyHouse.com). Subzero saline solution (189 L/6 birds) was prepared by dissolving salt for 4% (w/w) in tap water using a 389-L container. The solution was then placed overnight in a freezing room at −12°C, and the target temperature (−2.41°C) of the saline solution was achieved while agitating continuously as explained above.

### Carcass Chilling and Chilling Yield

Broiler carcasses, after evisceration and washing, were randomly assigned to 1 of the 4 chilling methods: 1) WIC at 0.5°C, 2) SSC in 4% NaCl/−2.41°C, 3) WIC-SSC, 4) Warm water immersion chill at 10°C-WIC (**WWIC-WIC**). In each replication, 2 medium size carcasses/treatment were selected to monitor the internal temperature of carcass breasts using a digital thermometer/logger (ThermoData Thermocouple Logger KTC, ThermoWorks, American Fork, UT). The carcasses in prechiller were transferred to a postchiller when their temperature reached 15°C, where the chilling was continued until the temperature reduced 4.4°C or lower. After chilling, carcasses were hung on a shackle for 5 min, weighted, and placed in a cooler at 4°C to 5°C before deboning at 3-h postmortem. Chilling yield was obtained using the formulation: (after-chill carcass weight/before-chill carcass weight) × 100%.

### Cooking Yield and Shear Force

Cooking yield was measured using the method of Metheny et al. (2019). Briefly, breast fillets were removed at 3-h postmortem and weighed for pre- and postcook weight according to USDA-Food Safety and Inspection Services (2001). The cooking yield was calculated using the formula: (postcook weight)/(precook weight) × 100. Shear force was determined by the razor-blade method of Cavitt et al. (2004). A texture analyzer (TAHDi, Texture Technologies Corp., Scarsdale, NY) was used after calibration with a 25-kg load cell. The razor blade (height, 24 mm; width, 8 mm) was set at 10 mm/s and penetrated to the depth of 22 mm as the test was triggered by a 10-g contact force. Shear values (N) were recorded as the maximum force obtained during the shear. Two shear forces were recorded in the quarter portion from the anterior tip of the breast.

### Color Measurements

Color values were measured on the surface of breast skin and breast fillets using Commission Internationale de l’Éclairage (**CIE**) L*, a*, and b* values where L* refers to lightness, a* to redness, and b* to yellowness. A chromameter (8-mm aperture, illuminant C; CR-400, Konika Minolta Sensing Inc., Osaka, Japan) was calibrated with a white plate (L*, 97.28; a*, - 0.23; b*, 2.43). Six readings of CIE L*, a*, and b* were measured on the surface with no blood or physical defects such as bruises, hemorrhages, full blood vessels, or tearing (Fletcher et al., 2000).

### Statistical Analysis

Data with 3 replications were statistically analyzed using the Proc MIXED procedure of SAS (SAS Institute, 2022) in a completely randomized design. If significance was determined (*P* < 0.05) in the model, dependent variable means were separated using the least significant difference procedure of SAS (*P* < 0.05; SAS Institute, 2022).

## RESULTS AND DISCUSSION

**Figure 1:**
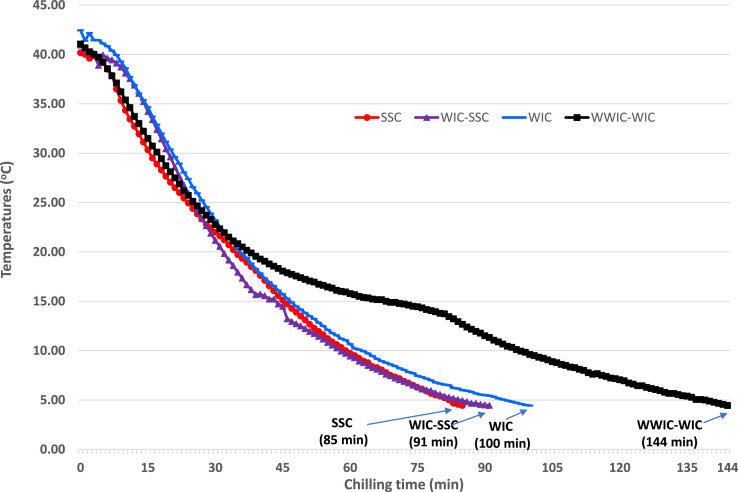
Temperature change profiles of broiler fillets during chilling* in water and brine solutions. *WIC, water immersion chilling using ice slurry at 0.5°C. SSC, subzero saline chilling using 4% NaCl at −2.41°C. WIC-SSC, WIC as a prechill and SSC as a postchill. WWIC-WIC, warm water immersion chill (WWIC) as a prechill and WIC as a postchill.

The average internal temperature of eviscerated carcass was 41.2°C that continuously decreased to the target temperature of 4.4 C or below during chilling. The carcasses in the SSC and WIC-SSC showed shorter chilling times (85–91 min) than those (100–144 min) of WIC and WWIC-WIC (Figure 1). The chilling trend of the SSC and WIC is in accordance with the previous study (Kang et al., 2022). In the poultry industry, carcasses are initially chilled in a prechiller at 7°C to 12°C for 10 to 15 min before entering a main chiller at 1°C to 4°C for 45 to 110 min, resulting in a total chilling time for 55 to 125 min depending on the carcass size (Sams and McKee, 2010). In our study, the combination of WWIC at 10°C and WIC at 0.5°C represents the current chilling method of the poultry industry. The chilling time (85 min) of single chiller (SSC) is faster than the current WIC (100 min), and the chilling time (91 min) of double chillers (WIC-SSC) is much faster than that (144 min) of the current WWIC-WIC (Figure 1).

**Figure 2:**
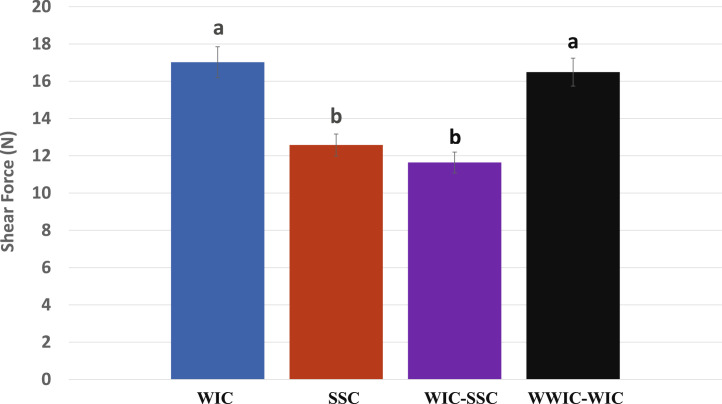
Effects of chilling methods on shear force^1^ of broiler fillets. ^a–b^Means with different superscript letters are different (*P* < 0.05). ^1^Number of observations in each chilling, *n* = 18. Chilling methods are the same as in Figure 1.

**Figure 3:**
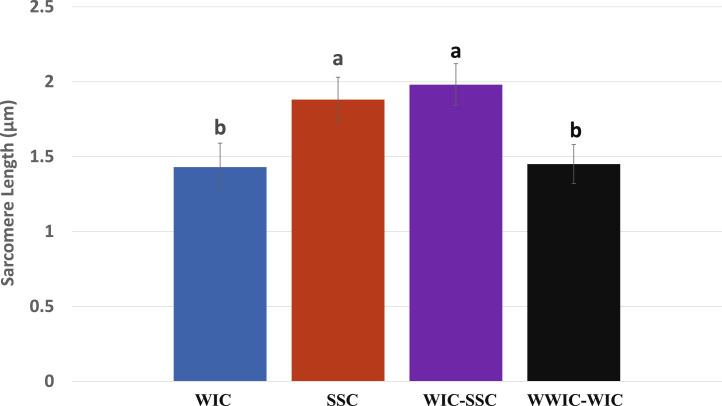
Effects of chilling methods on sarcomere length^1^ of broiler fillets. ^a–b^Means with different superscript letters are different (*P* < 0.05). ^1^Number of broilers in each chilling, *n* = 18. Chilling methods are the same as in Figure 1.

The cooked breast fillets of broilers in SSC and WIC-SSC showed lower shear forces than the WIC and WWIC-WIC (Figure 2). Subsequently, longer sarcomere lengths were observed in the muscle of SSC and WIC-SSC than the WIC and WWIC-WIC (Figure 3). These results indicated that the rapidly chilled carcasses produced more tender and less shrunken meat than those chilled slowly that were similarly observed by Lee et al. (2020) and Metheny et al. (2019). Dunn et al. (1995) reported that broilers chilled fast in water at 0°C and air at −12°C produced more tender meat than those chilled slowly in water at 10°C and air at 0°C. During the carcass chilling, muscles start to shrink near body temperatures (37°C–41°C) that is known as rigor shortening (or heat shortening) and further shrink at cold temperatures (0°C–5°C), known as cold shortening, whereas the least shortening was observed in the mid-temperature (14°C–19°C) between the 2 shrinking temperature zones (De Fremery and Pool, 1960; May et al., 1962; Khan, 1971; Papa and Fletcher, 1988). In comparison of the 2 shortening phenomena, rigor shortening at high temperatures (>20°C) in poultry was more severe than cold shortening at 0°C while opposite situation was observed in red meat (Husband, 1993; Bilgili et al., 2002).

There was no significant difference in muscle pH regardless of chilling methods (Table 1) that agrees with the previous results (Lee et al., 2020). Higher chilling yield was observed for the carcasses in the WIC-SSC than those in other chilling methods (*P* > 0.05) while no significant difference was observed in cooking yields (Table 1).

**Table 1:** Effect of chilling methods on carcass chilling yield and cooking yield.^1^

Parameter	WIC	SSC	WIC-SSC	WWIC-WIC	SE
pH	5.95	5.97	5.92	5.96	0.03
Chilling yield (%)	101.4^b^	101.5^b^	103^a^	101.8^b^	0.35
Cooking yield (%)	72.2	71.9	73.0	70.2	0.96

a–b Means with different superscript letters are different (*P* < 0.05).

1 Number of observations in each chilling, *n* = 18. Chilling methods are the same as in Figure 1. SE, standard error.

Chilling methods affect the visual appearance of broiler carcasses such as carcass color, surface wetness, and purge related wet pad (Huezo et al., 2007; Carroll and Alvarado, 2008; Jeong et al., 2011). There was no significant difference for CIE L* and a* on breast skin regardless of chilling methods while lower b* values were observed for SSC and WIC-SSC (*P* > 0.05) (Table 2). In the case of skinless breast surface, WWIC-WIC showed higher L* values than SSC, and intermediate values were seen for WIC and WIC-SSC. No significant difference was observed for a* value while higher b* values were seen in the WIC than in the SSC with intermediate values in the WIC-SSC and WWI-WIC (Table 2).

**Table 2:** Effect of chilling methods on the color^1^ of breast skin and fillet.

Parameter	WIC	SSC	WIC-SSC	WWIC-WIC	SE
Breast skin color					
L*	72.26	71.59	70.77	72.23	1.23
a*	2.76	2.29	2.85	3.05	0.41
b*	4.81^a^	0.16^b^	1.47^b^	3.93^a^	1.10
Breast fillet color					
L*	57.32^ab^	55.35^b^	56.39^ab^	58.68^a^	1.67
a*	1.33	1.37	1.50	1.48	0.46
b*	5.09^a^	3.94^b^	4.62^ab^	4.48^ab^	0.50

a–b Means with different superscript letters are different (*P* < 0.05).

1 Number of observations in each chilling, *n* = 108. Chilling methods are the same as in Figure 1. SE, standard error.

## CONCLUSIONS

The methods of SSC and WIC-SSC improved the chilling efficiency and meat tenderness more than WIC and WWIC-WIC with no significant difference in pH, cooking yield, and breast skin color except for b* values. Considering the fabrication of broilers at 2-h postmortem or less in the poultry processing plants, the faster chilling in SSC (85 min) and WIC-SSC (91 min) than the traditional chilling in WIC (100 min) and WWIC-WIC (114 min) is desirable. Considering the meat toughness upon deboning at 2-h postmortem after the traditional chilling, the improvement of meat tenderness using the new chilling is ideal. Similar research on turkey carcasses is expected to further improve chilling efficiency and meat quality.
